# Use of Streptococcus Salivarius K12 in a cohort of PFAPA patients

**DOI:** 10.3389/fped.2024.1370544

**Published:** 2024-07-25

**Authors:** Alessandra Spagnolo, Vincenzo Mileto, Adele Civino, Maria Cristina Maggio, Paolo Risso, Simona Sestito, Romina Gallizzi

**Affiliations:** ^1^ Pediatric Unit, Department of Health Science, Magna Grecia University of Catanzaro, Catanzaro, Italy; ^2^ Maternal and Infantile Department, PO “Vito Fazzi Hospital” Lecce, Lecce, Italy; ^3^ Department of Health Promotion, Maternal and Infantile Care, Department of Internal Medicine and Medical Specialties “G. D'Alessandro”, University of Palermo, Palermo, Italy; ^4^ Department of Health Sciences, University of Genoa, Genoa, Italy; ^5^ Pediatric Unit, Department of Medical and Surgical Sciences, Magna Graecia University, Catanzaro, Italy

**Keywords:** probiotic, PFAPA, Streptococcus Salivarius K12, children, prophylaxis, treatment, pharyngotonsillitis, fever

## Abstract

**Background:**

Periodic fever, aphthous stomatitis, pharyngitis and cervical adenitis syndrome (PFAPA) is the most common cause of periodic fever in childhood. Although PFAPA is generally a self-limited condition, it can have negative impact on child's and parents’ quality of life and family functioning. Our primary aim was to assess the potential effectiveness of Streptococcus Salivarius K12 (SSK12) in preventing febrile attacks in PFAPA patients. Secondary objectives included evaluating the effectiveness of SSK12 in mitigating the severity of febrile episodes seen as a statistically significant reduction in the episode duration, highest fever temperature reached during fever, in the frequency of each associated symptom, calculated in the six months before and after the start of therapy.

**Results:**

A total of 117 patients with PFAPA were evaluated using Marshall's criteria, modified by Thomas et al. and according to Eurofever/PRINTO classification criteria, aged 6 months to 9 years, with a median age at the onset of the disease of 2 years, treated with SSK12, since January 2021 to January 2023. Data were collected retrospectively. Before using SS K12, febrile episodes recurred on average every 26.1 ± 11.5 days, with a febrile episode duration of 4.1 ± 1.4 days. The highest fever temperature during the episode was 39.8 ± 0.7 °C. After six months of SS K12, febrile episodes recurred on average every 70 ± 53,1 days (*p* value <0.01), the mean lenght of febrile episodes was 3.3 ± 1.6 (*p* value <0.01) and the highest fever temperature reached during the febrile episode was 39.1 ± 1.1 °C (*p* value <0.01). We also documented a reduction in the frequency of exudative pharyngotonsillitis present in 72 vs. 103 patients (*p* value <0.01), oral aphthosis present in 47 vs. 80 patients (*p* value <0.01), lateral cervical lymphadenopathy in 45 vs. 83 (*p* value <0.01). Erythematous pharyngotonsillitis decreased in frequency but it was not statistically significant.

**Conclusions:**

The results of our study indicate that the use of SS K12 could be beneficial in decreasing febrile episodes related to PFAPA syndrome and its associated symptoms, potentially improving the quality of life in pediatric patients and decreasing the need for additional pharmacological therapies.

## Background

Periodic fever, aphthous stomatitis, pharyngitis and cervical adenitis syndrome (PFAPA) is the most prevalent cause of periodic fever in childhood. Characterized by recurrent fever, pharyngitis, oral aphthae and cervical lymphadenopathy, it was first described by Marshall et al. in 1987.

The diagnosis of PFAPA is distinguished by the periodic nature of the fever and the absence of upper respiratory tract (URTI) symptoms and sick contacts (1). This syndrome typically affects children under 5 years old and generally resolves spontaneously within 3–5 years from the onset of symptoms (2). Although PFAPA is a self-limiting condition, it can significantly impact a child's quality of life, daily activities, schooling, and family functioning (3).

The fever typically persists for 4–6 days, often escalating to 39–40.5 °C, and is unresponsive to antibiotic treatment. In addition, over 90% of children experience pharyngitis, up to 75% develop cervical adenitis, and up to 50% manifest oral aphthosis during episodes.

Other potential symptoms include headache, abdominal pain, arthritis, arthralgia, rash, and diarrhea (4).

Currently, a specific diagnostic assay for PFAPA remains unavailable, necessitating dependence on clinical evaluation supported by classification criteria (5). Typically, the condition resolves spontaneously in most children within a few years, primarily before puberty. However, approximately 20% of cases experience a recurrence of febrile episodes in adulthood subsequent to an initial remission (6).

Management of PFAPA symptoms often involves the administration of corticosteroids, nonsteroidal anti-inflammatory drugs (NSAIDs), or colchicine (7). Notably, febrile episodes in PFAPA exhibit a remarkable response to systemic corticosteroid therapy, with fever resolution occurring rapidly. This typical responsiveness aids in distinguishing PFAPA from other periodic fevers. Nevertheless, patients treated with corticosteroids often report a reduction in the duration of symptom-free intervals between episodes (8). Certain authorities recommend tonsillectomy (9) or tonsillotomy (10) as a curative measure.

Despite the absence of a standardized treatment regimen for PFAPA due to limited clinical trial data, the CARRA PFAPA group has developed a consensus treatment plan encompassing four arms: antipyretic therapy, corticosteroids, colchicine or cimetidine, and tonsillectomy.

Comparative analysis of outcomes across these treatment arms aims to identify approaches yielding optimal clinical responses for this syndrome (11).

The etiology of PFAPA remains unidentified with no single infectious agent definitively implicated.

Alterations in the entire microbiota and the intricate interplay between the microbiome and the underlying lymphoid tissue within the tonsils are hypothesized to hold sway over the activation of inflammatory responses.

Tonsillar microbiota may either serve as a precipitating factor for inflammatory processes in PFAPA or confer protection against inflammation in healthy children (12).

Additionally, a dysregulated interleukin (IL)-1 response is implicated in the pathogenesis of the disease (13, 14), and categorizing PFAPA as an autoinflammatory disorder (15).

Manthiram et al. demonstrated genetic and clinical parallels among recurrent aphthous stomatitis, PFAPA, and Behçet's disease, categorizing these conditions within a shared spectrum. Recurrent aphthous stomatitis is positioned at the mildest end, Behçet's disease at the most severe, and PFAPA occupies an intermediary position. Notably immunological profiling of peripheral blood samples reveals heightened monocyte and Th1 activation, evidenced by the up-regulation of IFN-induced transcripts and elevated levels of IL-12, IFN-γ, and Th1-associated chemokines during symptomatic episodes (16).

Furthermore, elevated levels of activated T lymphocytes, GM-CSF, G-CSF, and proinflammatory cytokines, including IL-1β, IL-6, and IL-8, have been documented during PFAPA attacks (17).

Streptococcus Salivarius K-12 (SS K12) emerges as an oral probiotic recently introduced for both pediatric and adult populations. SS K12, a prevalent colonizer of the oral cavity, produces two bacteriocins -salivaricin A2 and salivaricin B- that exert antagonistic effects against the growth of Streptococcus pyogenes, Streptococcus Pneumoniae, Haemophilus Influenza, Moraxella Catarrhalis, the most common bacterial strains implicated in the genesis of exudative pharyngotonsillitis and recurrent otitis media (18).

The evidence indicates that probiotics can effectively reduce both the frequency and duration of fever and are valuable in preventing occurrences of acute upper respiratory tract infections.

Manti et al. demonstrated the efficacy and the safety of Streptococcus Salivarius 24SMBc and Streptococcus oralis 89a in the short-term treatment of upper respiratory tract infections in children.

This therapeutic approach significantly reduced the incidence of fever, cough, bronchospasm, rhinorrhea, and otalgia episodes (19).

According to Guo et al., SS K12 has demonstrated efficacy in reducing upper respiratory tract infections in children, as well as in decreasing children's sick leave days or parents’ absence days from work, and diminishing the duration of antibiotic usage (20).

Furthermore, the utilization of probiotics may instigate alterations in the tonsillar microbiota, potentially averting inflammation in PFAPA patients (12).

A recent study delineated the advantageous impacts of two probiotic strains, Lactobacillus plantarum HEAL9 and Lactobacillus paracasei 8700:2, in a limited cohort of PFAPA patients by reducing the frequency of attacks through the anti-inflammatory mechanisms of the Lactobacilli or by modulating the tonsillar microbiota (4).

SS K12 has demonstrated potential effects in the realm of pharyngotonsillitis (21, 22) and it has also proven effective in reducing the levels of certain cytokines, such as IL-8, which play a role in the development of the inflammatory response and potentially in the pathogenesis of PFAPA (23).

Furthermore, SSK12 has been identified as beneficial in decreasing the overall number of febrile exacerbations associated with PFAPA syndrome and mitigating the severity of fever attacks in children (24).

The objective of our study was to assess the effects of SS K12 in a cohort of PFAPA patients to evaluate its effectiveness, assuming that the K12 strain, possessing anti-inflammatory properties, could offer benefits by diminishing both attack frequency and severity.

## Materials and methods

A total of 117 patients with PFAPA were diagnosed using Marshall's criteria (1), modified by Thomas et al. (25) and according to the Eurofever/PRINTO classification criteria (5).

Patients have been recruited through medical records of outpatient visits and follow-up visits since January 2021 to January 2023 at the Pediatric Rheumatology and Immunology Center, Magna Graecia University of Catanzaro. Data were collected retrospectively.

All patients were treated with paracetamol or NSAID or systemic corticosteroids (generally a single dose of betamethasone at the dosage of 0.1 mg/kg), during febrile episodes. The children were initially evaluated in our clinic and we prescribed SSK 12.

In the younger probands we excluded other forms of monogenic periodic fever syndromes using the next-generation sequencing (NGS) strategy.

Our primary aim was to assess the effectiveness of SSK12 in preventing febrile flares by measuring any statistically significant difference between the fever-free interval calculated in the six months preceding and following the start of therapy.

Secondary aims encompassed the evaluation of SSK12’s efficacy in reducing the severity of febrile episodes, seen as a statistically significant reduction in the episode duration and highest fever temperature reached during the febrile episode, calculated in the six months before (M0) and after (M6) the start of therapy and a statistically significant reduction in the frequency of each associated symptom, calculated in the six months before and after the start of therapy.

To determine the frequency of symptoms, the appearance of symptoms in the most febrile episodes was assessed. Symptoms were assessed by asking parents careful questions.

Administration of SSK12 occurred at the standard dosage of 1 billion colony-forming units per tablet of SS K12: every day before sleeping, the patient was given a tablet and asked to take it without biting or swallowing.

We also analyzed demographic, clinical, laboratory, instrumental, and therapeutic data accessible for the patients. Moreover, prior to inclusion, all parents furnished written consent, authorizing the utilization of their data for scientific endeavors.

JMP v16 has been used to perform all the analysis and statistics. We first conducted the Shaprio-Wilk Test to assess the normality of our data. Upon finding significant deviation from normal distribution, we proceeded with the Rank Sum Wilcoxson for matched pairs to detect statistical differences before and after treatment with SS K12. Statistical significance was defined as at *p*-value <0.01.

## Results

A total of 117 patients with PFAPA were evaluated using Marshall's criteria) (1), modified by Thomas (25) et al. and according to the Eurofever/PRINTO classification criteria (5), including 57 females (48.7%) and 60 males (51.3%), aged 6 months to 9 years (102 patients aged 0–3 years old, 10 aged 3–6 years and 5 aged 6–9 years). At disease onset, the median age was 2 years (0.5–9). 73 (62.4%) patients had a family history of recurrent fevers in childhood and 62 (52.9%) patients had a parent or first-degree family member who was undergoing tonsillectomy or adenoidectomy.

Symptoms typically began at around 2 ± 1.6 years. The mean age of enrollment in the study was 4.3 ± 2.7 years.

Before using SS K12, febrile episodes recurred on average every 26.1 ± 11.5 days, with a febrile episode duration of 4.1 ± 1.4 days. The peak fever temperature observed in children during the episode was 39.8 ± 0.7 °C (Table 1).

**Table 1:** Quantitative variables during six months using SS-K12.

Variable	Baseline	M6	*p* value
Highest fever temperature (°C)	39.8 ± 0.7	39.1 ± 1.1	2.8^−13^
Duration of fever (days)	4.1 ± 1.4	3.3 ± 1.6	8.9^−12^
Intervals between attacks (days)	26.1 ± 11.5	70 ± 53.1	1.3^−12^
Erythematous pharyngotonsillits	82	72	0.85
Exudative pharyngotonsillits	103	72	5.8^−6^
Oral aphtosis	80	47	1.6^−5^
Lateral cervical lymphadenopathy	83	45	6.4^−7^

M0, baseline. M6, after six months using SSK12.

After six months of SS K12, febrile episodes recurred on average every 70 ± 53,1days, the average duration of febrile episodes was 3.3 ± 1.6 and the highest fever temperature reached during the febrile episode was 39.1 ± 1.1 °C. (*p* = 1.3^−12^) at diagnosis patients during each febrile episode had the following associated symptoms: erythematous pharyngotonsillitis (82), exudative pharyngotonsillitis (103), oral aphthosis (80), lateral cervical lymphadenopathy (83).

We tested for normality distribution the following variabiles: Highest fever temperature, duration of fever days, interval between attacks (days) with Shaprio Wilk Test. All the variables showed a non normal distribution: Highest fever temperature M0 *p* = 0.0012, M6 *p* = 0.0009; duration of fever days M0 *p* = 4.1^−4^, M6 *p* = 1.9^−4^; interval between attacks (days) M0 *p* = 6.3^−11^, M6 *p* = 1.1^−10^.

We also documented a reduction in the frequency of exudative pharyngotonsillitis present in 72 (61.5%) vs. 103 (88%) patients (*p* value <0.01), of oral aphthosis present in 47 (40.2%) vs. 80 (68.4%) patients (*p* value <0.01), lateral cervical lymphadenopathy in 45 (38.5%) vs. 83 (71%) (*p* value <0.01).

Erythematous pharyngotonsillitis decreased in frequency: 72 (61.5%) vs. 82 (70.08%), which was not statistically significant (Table 1).

This treatment was been well accepted by children and their parents, as well as for its safety.

It was well tolerated, with no adverse events reported.

## Discussion

The exact prevalence of PFAPA remains elusive, emerging evidence suggests a higher occurrence rate than previously estimated, with indications that it could be the predominant recurrent autoinflammatory fever syndrome among children. The reported incidence of PFAPA is approximately 2.3 per 10,000 children up to the age of 5 years as described in a Norwegian study (26).

Although PFAPA is generally perceived as relatively benign, many patients experience persistent symptoms over years. However, PFAPA patients commonly face non-medical challenges, such as missed school days due to febrile attacks, which can have a considerable impact on both the family and the psychological well-being of the affected child. Grimwood, Kone-Paut et al. suggest that the quality of life of PFAPA children is significantly compromised, with notable effects on psychosocial functioning and fatigue (27).

This study primarily aimed to assess the effectiveness of the probiotic SS K12 in treating PFAPA patients.

Currently, probiotics are increasingly utilized for the prevention and treatment of various diseases.

The main goal of probiotic treatment is to promote intestinal health. They are described as being able to counteract constipation, diarrhea or irritable bowel syndrome (28).

Probiotic therapy can also be targeted for a non-intestinal benefit. Examples of such applications include efforts to prevent or treat gynecological and/or urological conditions such as vaginitis, vaginosis, or cystitis, especially in patients with recurrent forms. Recent trials have indicated that probiotics, specifically Lactobacillus and Bifidobacterium species, may be effective in preventing episodes of acute upper respiratory tract infections (29). However, only SS K12 has demonstrated potential benefits in the context of pharyngotonsillitis (21, 22). Many studies have shown that SS K12, when administered in the form of oral dissolvable tablets, possesses a favorable safety profile and can establish persistent colonization within the upper respiratory tract (30, 31).

His probiotic's mechanism of action includes mitigating the risk of oral colonization by Streptococcus pyogenes. Similar antagonistic effects have been observed against other strains, which are susceptible to the same two lantibiotics and are associated with conditions such as acute otitis media and halitosis (32).

Recent findings suggest that oral administration of the SSK12 strain not only colonizes the oropharynx, releasing two salivaricins that act as antagonists against certain pathogenic strains but also, through a molecular mechanism that is not yet fully elucidated, reduces plasma levels of IL-8 and increases the concentration of salivary interferon-γ (33).

These modulations provide a rational explanation for its anti-inflammatory activity, complementing its inherent antibacterial properties.

In a recent study, Ozen et al. assessed the response to other probiotic treatment in PFAPA patients. Their patients received a probiotic formulation containing a combination of two lactobacilli, resulting in a reduction in attack frequency. This observation suggests that probiotic strains Lactobacillus plantarum HEAL9 and Lactobacillus paracasei 8700:2 could offer benefits to PFAPA patients by decreasing the frequency of attacks (4).

Manti et al. conducted a study aimed at evaluating the effectiveness and safety of Pidotimod in treating children with PFAPA syndrome. Their findings suggested that high doses of Pidotimod could serve as an effective and safe means to reduce PFAPA attacks in children. In their investigation, 19 out of 22 children experienced favorable outcomes following Pidotimod administration, manifesting in a notable reduction in fever frequency, pharyngitis, aphthous stomatitis, as well as the necessity for betamethasone usage (34).

La Torre et al. also show how SSK12 is effective in reducing the total number of febrile exacerbations related to PFAPA syndrome per year, reducing the duration of a single febrile episode, lowering body temperature during the episode, and reducing the use of other drugs such as corticosteroids in children (24).

In our patients prophylaxis with SS K12 delayed the time between febrile episodes, it reduced their duration and also the maximum body temperature during fever. Furthermore, during treatment the frequency of clinical signs was reduced suggesting how the attack severity decreased significantly during probiotic administration.

The findings of this longitudinal study, despite its limitations such as the lack of a control group and the wide range of age, suggest that oral formulations containing SS K12 could represent a valuable option for both preventing and managing fever attacks in PFAPA patients.

## Conclusion

Our evaluation has concluded that SSK12 is effective in reducing febrile episodes related to PFAPA syndrome and its associated symptoms. It would therefore be able to improve the quality of life, in pediatric patients and their families, reducing the number of school absences, parents missing work and the necessity of additional pharmacological therapies. However, further studies are needed to support these preliminary data.

All methods were performed in accordance with the ethical standards as laid down in the Declaration of Helsinki and its later amendments or comparable ethical standards.

## Data Availability

The raw data supporting the conclusions of this article will be made available by the authors, without undue reservation.
